# Comparative analysis of clinicopathological characteristics of central necrotizing breast cancer and basal cell-like breast cancer

**DOI:** 10.3389/fonc.2023.915949

**Published:** 2023-03-31

**Authors:** Li Ding, Wang Kun, Wenjing Xu, Shaohua Chen, Zhaogen Cai

**Affiliations:** ^1^ Department of Pathology, The First Affiliated Hospital of Bengbu Medical College, Bengbu, China; ^2^ Department of Pathology, Mengcheng Hospital of Traditional Chinese Medicine, Bozhou, Anhui, China; ^3^ Department of Pathology, Bengbu Medical College and The First Affiliated Hospital of Bengbu Medical College, Bengbu, China

**Keywords:** centrally necrotizing carcinoma of the breast, basal-like breast cancer, BRCA1, HIF-1α, VEGF

## Abstract

**Purpose:**

This study aims to compare the clinicopathological and immunohistochemical characteristics of centrally necrotizing carcinoma of the breast (CNC) and basal-like breast cancer (BLBC), as well as to analyze the characteristics of the molecular typing of the CNC.

**Methods:**

The clinicopathological features of 69 cases of CNC and 48 cases of BLBC were observed and compared. EnVision immunohistochemical staining was performed to detect the expressions of hypoxia-inducible factor 1α (HIF-1α), breast cancer susceptibility gene 1 (BRCA1), and vascular endothelial growth factor (VEGF) in CNC and BLBC.

**Results:**

The age of the 69 patients ranged from 32 to 80 years, with an average of 54.55 years. Gross examination showed that most tumors were well-defined single central nodules with a diameter of 1.2~5.0 cm. Microscopically, there is a large necrotic or acellular area in the center of the tumor, mainly composed of tumor coagulative necrosis with varying degrees of fibrosis or hyaline degeneration. A small amount of cancer tissue remained in the form of a ribbon or small nest around the necrotic focus. Among 69 cases of CNC, the proportion of basal cell type (56.5%) was significantly higher than that of lumen type A (18.84%), lumen type B (13.04%), HER2 overexpression (5.8%), and nonexpression (5.8%). A total of 31 cases were followed up for 8~50 months, with an average of 33.94 months. There have been nine cases of disease progression. When compared to BLBC, there were no significant differences in BRCA1 and VEGF protein expression in response to CNC (*p* > 0.05), but there were significant differences in protein expression in HIF-1α (*p* < 0.05).

**Conclusion:**

The molecular typing of CNC showed that over half of those were BLBC. No statistically significant difference in the expression of BRCA1 was observed between CNC and BLBC; thus, we predict that targeted therapy for BRCA1 in BLBC may also have considerable effects in CNC patients. The expression of HIF-1α is significantly different in CNC and BLBC, and perhaps HIF-1α can be used as a new entry point to distinguish between the two. There is a significant correlation between the expression of VEGF and HIF-1α in BLBC, and there was no significant correlation between the expression levels of the two proteins in CNC.

## Introduction

Centrally necrotizing carcinoma of the breast (CNC) has attracted widespread attention from scholars due to its particular pathological morphology and its similar immunological and morphological characteristics to BLBC, and in the progress of the disease, CNC also has high value-added activity and invasiveness. In 2001, Jimenez officially named CNC (1); prior to this event, Hasebe in 1997 and Tsuda in 1999, respectively, reported a group of central acellular invasive ductal carcinoma (2, 3). However, the breast cancer defined by the above three reports essentially has similar clinicopathological features. The concept of CNC has not yet been recognized by the WHO, and there are only more than 10 reports on CNC at home and abroad. The current perceptions of CNC are insufficient in both clinical and histological aspects. The BLBC subtype of CNC reported by Yu et al. in 2009 and Zhang et al. in 2015 accounted for 63.6% and 50.7% (4, 5), respectively. Therefore, we argue that there exists a close connection between CNC and BLBC. Based on the above, this study intended to observe the clinicopathological features of the CNC and BLBC, detect BRCA1 and hypoxia-inducible factor 1α (HIF-1α) and vascular endothelial growth factor (VEGF) expression differences, and further analyze the correlations between the two.

## Materials and methods

### Material collection

Specimens from 69 cases of CNC with necrosis areas of >70%, 48 cases of BLBC confirmed by immunohistochemistry, and 36 cases of invasive ductal carcinoma served as controls were collected from the Department of Pathology, First Affiliated Hospital of Bengbu Medical College. All patients had received no prior treatment before surgery; there was no history of other malignant tumors, and all specimens were obtained from the modified radical mastectomy.

The experimental sample obtained was approved by the ethics committee of the First Affiliated Hospital of Bengbu Medical College, and informed consent was obtained from all the patients.

### Reagents

Primary antibodies ER (clone: 1D5), PR (clone: MX009), EGFR (clone: SP125), VEGF (clone: VG1), Ki-67 (clone: MIB-1), and P53 (clone: BP52-12) were from Fuzhou Maixin: Fuzhou, Fujian, China. HER-2 (clone: IHC042) and CK5/6 (clone: IHC556) were from Shenzhen Aibimeng: Shenzhen, Guangdong, China. BRCA1 (clone: MS110) and HIF-1α (clone: EP1215Y) were from Abcam USA: Hangzhou, Zhejiang, China. Auxiliary reagents (PBS buffer, DAB chromogenic solution, hydrogen peroxide solution, differentiation solution, xylene, various concentrations of alcohol and absolute alcohol, secondary antibody, hematoxylin stain) were purchased from Fuzhou Maixin.

### Method

All specimen tissues were fixed with 4% formaldehyde and serially sectioned at 4 μm in thickness, and immunohistochemical staining was performed with the two-step EnVision method. The operating procedure was carried out strictly according to the operating instructions of the kit. The known positive tissues of other breast cancer patients were used as positive controls, while the PBS buffer served as a negative control. Senior pathologists evaluated the histopathological features of all cases based on standard pathology methods by observing hematoxylin–eosin‐stained paraffin sections under the microscope. Clinicopathological staging and grading of all cases were performed according to the eighth edition of the American Joint Committee on Cancer (AJCC) staging system.

### Interpretation criteria for immunohistochemistry results

Expression of ER, PR, and HER2 was assessed according to the new American Society of Clinical Oncology/College of American Pathologists (ASCO/CAP) guidelines (6, 7). The positive expression products of CK5/6 and VEGF are located in the cytoplasm, and the positive expression products of EGFR are located in the cytoplasm and cell membrane. Staining was assessed on a semi-quantitative system: the intensity of staining was defined as 0, no staining; 1+, light yellow; 2+, brownish yellow; and 3+, brown. Scoring for the percentage of positive cells: negative was 0 points, less than 10% was 1 point, 11%–50% was 2 points, 51%–75% was 3 points, and more than 75% was 4 points. The total score of each section was defined as the product of the stained area score and the staining intensity score; a score of ≤3 was negative, while 3–12 was positive. The BRCA1 expression product is mainly localized in the nucleus, and the scoring system of Yoshikawa et al. is used (8), namely: 0% nuclear staining (deletion) is 0 points, <20% nuclear staining (reduce staining) is 1 point, 20%–80% nuclear staining is 2 points, >80% nuclear staining is 3 points, 0 points, and 1 point, depending on it, are considered negative, and those with a score of 2 and 3 are considered positive. The HIF-1α expression product is in the cytoplasm and/or nucleus, and tumors were defined as positive when ≥1% of tumor cells show immunoreactivity (9). The positive expression products of P53 are located in the nucleus, and tumors were defined as positive when >10% of tumor cells show immunoreactivity. The positive expression products of Ki-67 are located in the nucleus, and it is defined as a high expression when the proportion of positive cells is more than 30%, otherwise, it is a low expression.

### Molecular typing of breast cancer

According to Carey et al., Breast Cancer Molecular Classification Standards (10), 69 cases of CNC were classified as follows: luminal A is defined as ER+ and/or PR+ or HER-2−; luminal B is defined as ER+ and/or PR+ or HER-2+; BLBC is defined as ER−, PR−, and HER-2−, and any of the basal-like markers positive (CK5/6 and EGFR); HER-2-overexpressing type is negative for ER and PR, and HER-2 is strongly positive; the null phenotype is that none of the abovementioned markers are expressed.

### Case follow-up

Follow-up data were collected through medical records and telephone calls. The time to overall survival is defined as the time from diagnosis to death or the last follow-up. Disease progression was defined as tumor recurrence, metastasis, and death due to the disease.

### Data analysis

Data statistics and analysis were conducted using SPSS 26.0. The Chi-square test or Fisher exact probability method was utilized for analyzing qualitative data.

## Result

### Clinical data

#### CNC

The mean age was 54.55 ± 9.683, with a range of 32~80 years. On microscopic examination, 25 cases were lymph node positive and 44 cases were lymph node negative. Follow-up data were available for 31 patients included, with a mean length of follow-up of 33.94 ± 11.550 months (range, 8–50 months). Disease progression was found in nine patients under follow-up, including five deaths and five remote metastases (including two cases (2.90%) of the lung, one case (1.45%) of the brain, and two patients (2.90%) with coincident liver metastases and bone metastases).

#### BLBC

**Figure 1:**
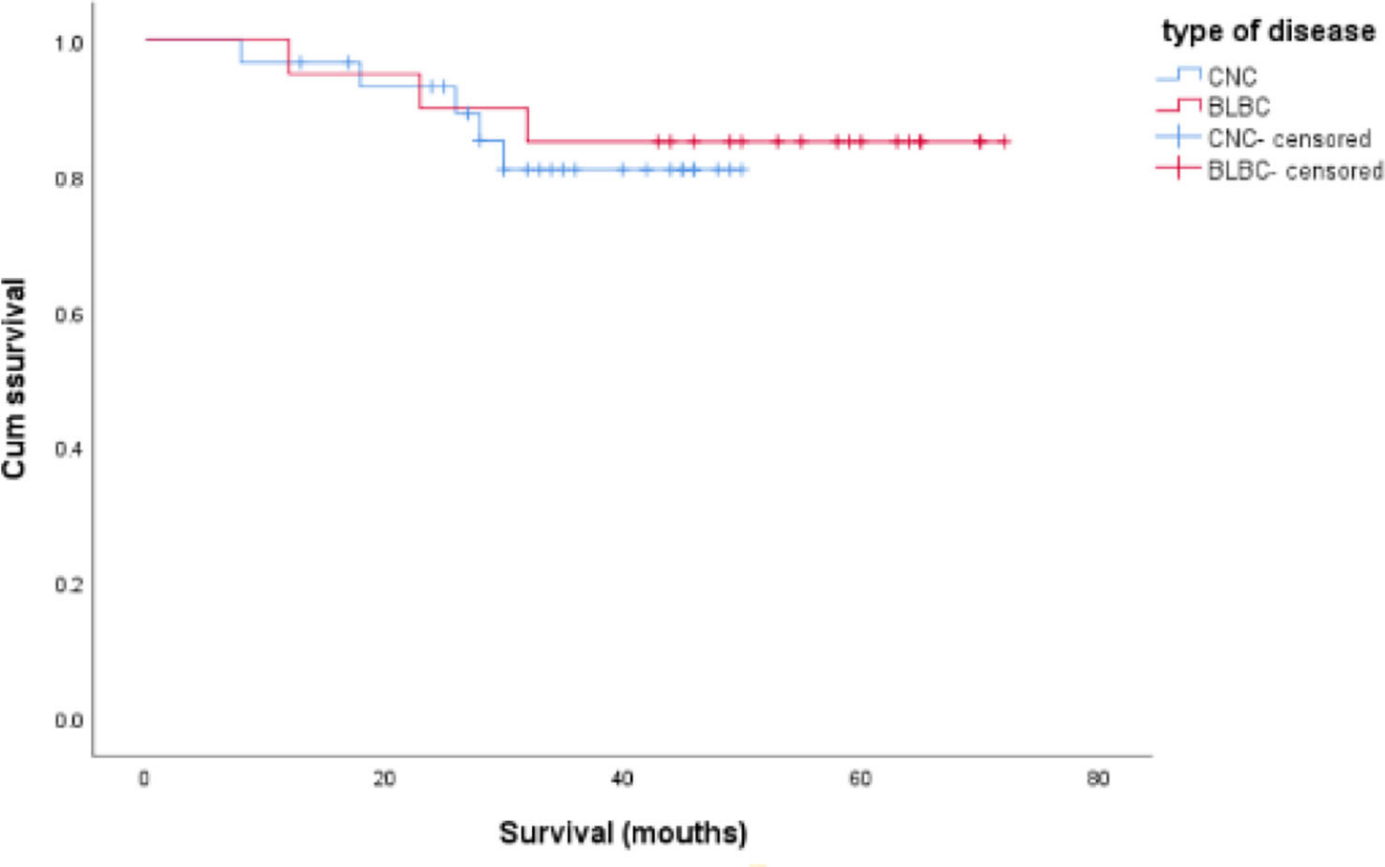
Comparison of overall survival time between CNC and BLBC patients, and the data analysis was not statistically significant (log-rank = 0.125, *p* > 0.05).

The mean age was 53.83 ± 11.372, with a range of 27–78 years. On microscopic examination, 21 cases were lymph node positive and 27 cases were lymph node negative. Follow-up data were available for 20 patients included with a mean length of follow-up of 52.65 ± 15.955 months (range, 12~72 months). Disease progression was found in seven patients under follow-up, including three cases (6.25%) of deaths, one case (2.08%) of brain metastasis, and three cases (6.25%) of sternal metastases. The overall survival time is shorter for CNC than BLBC, but the difference did not exhibit statistical significance (log-rank = 0.125, *p* > 0.05, **Figure 1**). Comparison of the clinicopathological features of CNC and BLBC (as depicted in **Table 1**).

**Table 1:** Comparison of clinicopathological parameters between CNC and BLBC.

Clinicopathological parameters	Number of CNC group (%)	Number of BLBC group (%)	*χ* ^2^	*p*-value
Age (year)				
≤50	27 (39.1)	22 (45.8)	0.523	0.470
>50	42 (60.9)	26 (54.2)		
T stage				
≤2 cm	28 (40.6)	17 (35.4)	2.644	0.260
2–5 cm	41 (59.4)	29 (60.4)		
>5 cm	0 (0.0)	2 (4.2)		
Histological grading				
G1+G2	6 (8.7)	3 (6.3)	0.238	0.735
G3	63 (91.3)	45 (93.8)		
Lymph node status				
Metastasis	25 (36.2)	21 (43.8)	0.671	0.413
Nonmetastasis	44 (63.8)	27 (56.3)		
Clinical stage				
I	24 (34.8)	11 (22.9)	1.991	0.370
II	27 (39.1)	21 (43.8)		
III	18 (26.1)	16 (33.3)		
Pathological stage				
I	29 (42.0)	11 (22.9)	4.600	0.100
II	23 (33.3)	21 (43.8)		
III	17 (24.6)	16 (33.3)		
ER				
Negative	48 (69.6)	47 (97.9)	14.903	<0.001
Positive	21 (30.4)	1 (2.1)		
PR				
Negative	53 (76.8)	47 (97.9)	10.153	0.001
Positive	16 (23.2)	1 (2.1)		
HER-2				
Negative	51 (73.9)	45 (93.8)	7.564	0.006
Positive	18 (26.1)	3 (6.3)		

### Histopathological features

#### Gross morphology of the CNC

**Figure 2:**
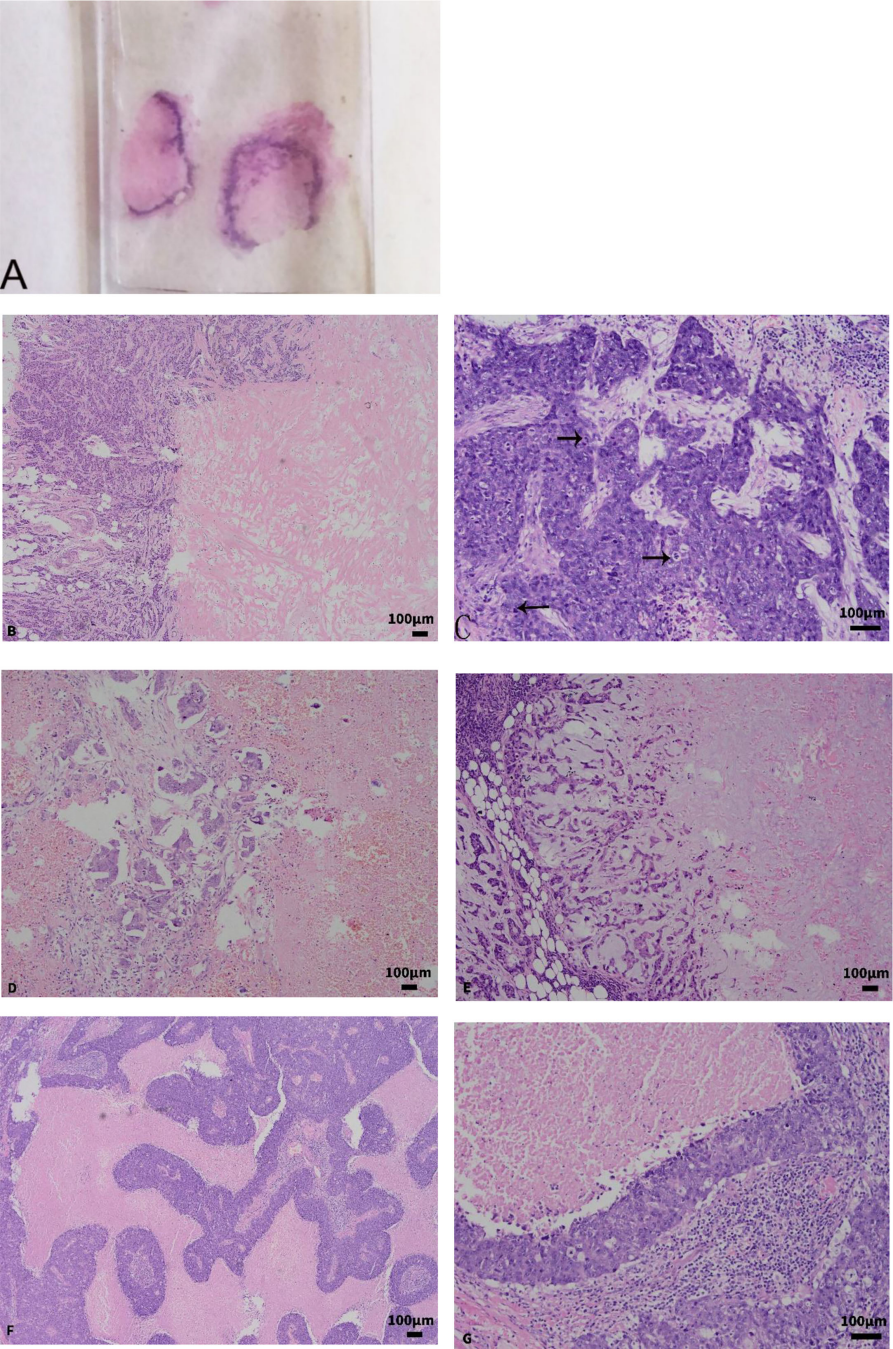
**(A)** Gross observation of the section; the center of the tumor is an extensive red-stained area surrounded by a blue-stained linear tissue. **(B)** The central red-stained area was mainly fibrous collagen tissue, glass-like and scar-like tissues, arranged in bundles and grids (×100). **(C)** Cancer tissue is more atypia and has more mitotic figures (×200). **(D)** The focally invasive micropapillary carcinoma was found in residual cancer tissue (×100). **(E)** Myxoid and chondroid stroma was found in residual cancer tissue (×100). **(F, G)** BLBC showed map-like or zonal necrosis (**F** ×100; **G** ×200).

In total, 67 of the 69 CNC were single nodules, and two were two nodules of the ipsilateral breast (mean nodule diameter was 2.55 ± 0.837 cm with a range of 1.2 to 5.0 cm). The boundary of most tumors was clear, and only four cases had unclear borders. Visual observation of HE sections revealed that the center of the tissue block is extensively red-stained, and the surrounding blue-stained area is distributed in a banding morphology around the central red-stained area (**Figure 2A**).

#### Microscopic examination

The central extensive cell-free zone is necrosis or fibrillar collagen, and the marginal zone is the cancerous cell distributed in bands. There is a lack of new granulation tissues and a fibrous tissue-like transitional zone between the two. Three distinct necrotic morphology were visible: (1) In 50 cases of CNC, coagulative tumor cell necrosis, granular morphology was seen in the necrotic area, the outline of cancer cells still existed, and nest-like, bundled collagen tissue and hyaloid or scar-like tissue interspersed in it. (2) The central cell-free area of the CNC in 19 cases was mainly fibrous collagen tissue, glass-like and scar-like tissues, arranged in bundles and grids (**Figure 2B**), with a small amount of tumor cell residual ghosting in the red-stained background. (3) Nine cases were infarctions; the outline of the tissue still existed, and there was no significant collagen fibril organization. The peripheral residual cancer cells display a band-like distribution, and they also exhibited the following significant atypia compared to normal cells: lack of glandular differentiation, high histological grade, and common mitotic figures (**Figure 2C**). In the 69 CNC, 63 cases (91.3%) of residual cancer tissue exhibited invasive ductal carcinoma grade III, six cases (8.7%) exhibited invasive ductal carcinoma grade II, 16 cases combined with ductal carcinoma *in situ*, and three cases with focally invasive micropapillary carcinoma (**Figure 2D**). The residual tumor stroma was myxoid and chondroid in 14 cases (**Figure 2E**), 39 cases had a large amount of lymphocyte infiltration, 1 case was mainly plasma cells, and 13 cases were accompanied by calcification.

#### Gross morphology of the BLBC

The 48 cases of BLBC were all single nodules, with an average diameter of 2.81 ± 1.194 cm (range, 1.2–7.0 cm), and the boundaries were unclear. There was no specific characteristic identified by macroscopic observations.

#### Microscopic examination

In total, 29 cases were accompanied by necrosis, of which nine cases showed map-like or zonal necrosis (**Figures 2F, G** **)**, three cases showed fibrous collagen in the center of the tumor, two cases had infarction, and the rest were punctate necrosis and the necrotic area was less than 30%. In the 48 BLBC, 46 cases (95.83%) were presented as invasive ductal carcinoma grade III, and two cases (4.17%) were invasive ductal carcinoma grade II. Only two cases were accompanied by ductal carcinoma *in situ*, one case had mucinous stroma in the background, 18 cases had lymphocytic infiltration, and calcification was observed in three cases. In CNC and BLBC, four cases and two cases of intravascular cancer embolus, and one case of nerve invasion were found, respectively. CNC and BLBC have statistical differences in the presence of ductal carcinoma *in situ* and mucinous stroma (*p* < 0.05) (**Table 2**). There is no significant difference between CNC and BLBC in the presence of interstitial lymphocyte infiltration, while there are statistical differences between the CNC and control groups (*p* < 0.05). The other characteristics were not significantly different between CNC, BLBC, and the control group (*p* > 0.05).

**Table 2:** Comparative analysis of cancer stroma between CNC and BLBC, and between CNC and control group.

Features of cancer stroma	Number of CNC group (%)	Number of BLBC group (%)	*χ* ^2^	*p*-value	Control group (%)	*χ* ^2^	*p*-value
Ductal carcinoma *in situ*	16 (23.19)	2 (4.17)	10.153	0.001	0 (0.00)	9.849	0.002
Lymphocyte infiltration in the cancer stroma	39 (56.52)	18 (37.5)	1.151	0.283	8 (22.22)	12.183	<0.001
Myxoid tumor stroma	14 (20.29)	1 (2.08)	8.395	0.004	1 (2.78)	5.925	0.015
Invasive micropapillary carcinoma	3 (4.35)	0 (0.00)	2.881	0.090	0 (0.00)	2.170	0.141
Calcification	13 (18.84)	3 (6.25)	3.801	0.059	6 (16.66)	0.075	0.784
Intravascular cancer embolus	4 (5.80)	2 (4.17)	0.135	0.713	2 (5.56)	0.476	0.490
Nerve invasion	1 (1.45)	1 (2.08)	0.068	0.795	1 (2.78)	0.223	0.636

### Immunohistochemical staining results

#### The expression of ER, PR, and HER-2 between CNC and BLBC

ER was positive in 30.43% of CNC and 2.08% of BLBC, PR was positive in 23.19% of CNC and 2.08% of BLBC, and HER-2 was positive in 26.09% of CNC and 6.25% of BLBC. The proportions of triple-negative breast cancer in CNC and BLBC were 53.6% and 93.8%, respectively. The results of the comparison are summarized in **Table 1**.

#### Molecular typing results

**Figure 3:**
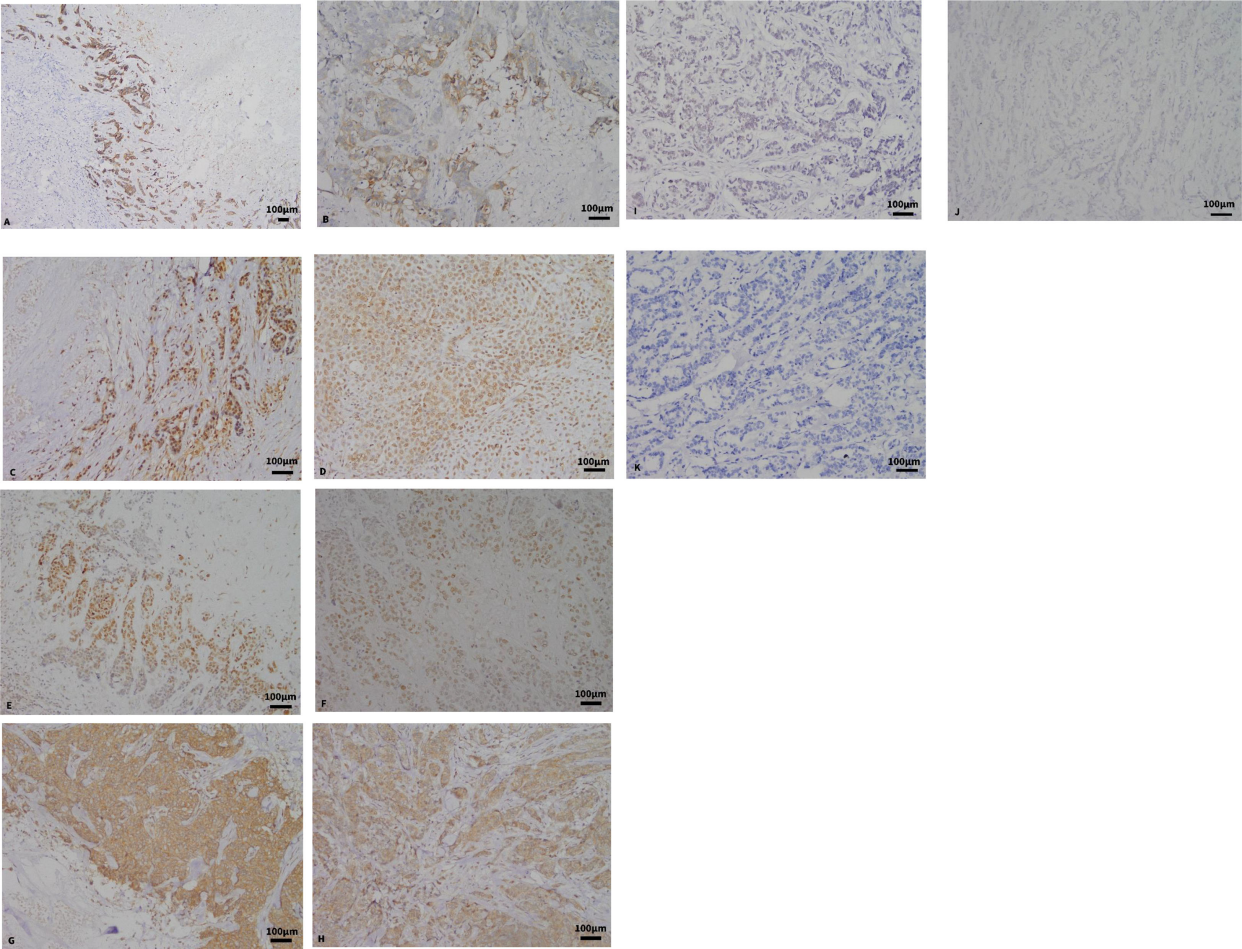
**(A, B)** Expression of CK5/6 and EGFR in CNC by immunohistochemical staining (×100, ×200). **(C)** The expression of BRCA1 in CNC. **(D)** The expression of BRCA1 in BLBC (×200). **(E)** The expression of HIF-1α in CNC (×200). **(F)** The expression of HIF-1α in BLBC (×200). **(G)** The expression of VEGF in CNC (×200). **(H)** The expression of VEGF in BLBC (×200). **(I)** The expression of BRCA1 in the control group. **(J)** The expression of HIF-1α in the control group. **(K)** The expression of VEGF in the control group.

There were 66.67% of CNC-expressed basal cell markers, of which 34 were positive for CK5/6 and 35 were positive for EGFR (**Figures 3A, B** **)**.

#### Molecular typing results

Of a total of 69 CNC, there were 13 (18.84%) luminal A, nine (13.04%) luminal B, four (5.8%) HER-2-overexpressing type, four (5.8%) null phenotype, and 39 (56.52%) BLBC. It can be seen that the proportion of BLBC subtype in CNC is the highest, and more than half of CNC express basal cell markers.

#### Analysis of the results of BRCA1, HIF-1α, and VEGF protein expressions

The positive expression rates of BRCA1 in CNC and BLBC were 46.4% and 39.6%, respectively (**Figures 3C, D** **)**. No statistical difference was observed between CNC and BLBC (*p* > 0.05), but there was a significant statistical difference between CNC and the control group (*p* < 0.05) (as depicted in **Table 3**). BRCA1 expression was not correlated with any clinicopathological parameters in CNC patients (*p* > 0.05), and the expression in BLBC was only associated with tumor size (*p* < 0.05).

**Table 3:** Comparison of differences in BRCA1 expression between CNC and BLBC and between CNC and control group.

Groups	Number of cases	BRCA1 expression		*χ* ^2^	*p*-value
		Positive	Negative		
CNC	69	32 (46.4%)	37 (53.6%)	–	–
BLBC	48	19 (39.6%)	29 (60.4%)	0.531	0.466
Control group	36	26 (72.2%)	10 (27.8%)	6.391	0.011

CNC, centrally necrotizing carcinoma of the breast.

BLBC, basal-like breast cancer.

The positive rates of HIF-1α expression in CNC and BLBC were 84.1% and 58.3%, respectively (**Figures 3E, F** **)**. Statistical differences were found between CNC and BLBC (*p* < 0.05) and between CNC and the control group (*p* < 0.05) (as depicted in **Table 4**). Analysis results showed that the expression of HIF-1α in CNC was only correlated with histological grade (*p <* 0.05), and it was related to lymph node metastasis, clinical stage, and pathologic stage in BLBC (*p* < 0.05).

**Table 4:** Comparison of HIF-1α expression differences between CNC and BLBC and CNC and control group.

Groups	Number of cases	HIF-1α expression		*χ* ^2^	*p*-value
		Positive	Negative		
CNC	69	58 (84.1%)	11 (15.9%)	–	–
BLBC	48	28 (58.3%)	20 (41.7%)	9.619	0.002
Control group	36	19 (52.8%)	17 (47.2%)	11.837	0.001

CNC, centrally necrotizing carcinoma of the breast.

BLBC, basal-like breast cancer.

The positive rates of VEGF expression in CNC and BLBC were 63.8% and 52.1%, respectively (**Figures 3G, H** **)**. No statistical differences were found between CNC and BLBC (*p* > 0.05) and CNC and control group (*p* > 0.05) (as depicted in **Table 5**). Analysis results showed that the expression of VEGF in CNC was only correlated with lymph node metastasis (*p <* 0.05), and it is related to lymph node metastasis, clinical stage, and pathologic stage in BLBC (*p* < 0.05) (**Figures 3I–K** shows the negative expression of BRCA1, HIF-1α and VEGF in the control group).

**Table 5:** Comparison of VEGF expression differences between CNC and BLBC and CNC and control group.

Groups	Number of cases	VEGF expression		*χ* ^2^	*p*-value
		Positive	Negative		
CNC	69	44 (63.8%)	25 (36.2%)	–	–
BLBC	48	25 (52.1%)	23 (47.9%)	1.597	0.206
Control group	36	22 (61.1%)	14 (38.9%)	0.072	0.789

CNC, centrally necrotizing carcinoma of the breast.

BLBC, basal-like breast cancer.

In the CNC group, the positive rate of VEGF was significantly higher in the BRCA1-negative group (78.38%) than in the BRCA1-positive group (46.88%), and the expression of VEGF and BRCA1 showed an inverse correlation (*r* = −0.327, *p* < 0.05). The positive rate of VEGF was significantly higher in the HIF-1α-positive group (67.24%) than the HIF-1α-negative group (45.46%), but there is no correlation between VEGF and HIF-1α (*r* = 0.166, *p* > 0.05) (as depicted in **Tables 6**, **7**).

**Table 6:** Correlation analysis of BRCA1 and VEGF in CNC and BLBC and CNC and control group.

Groups	BRCA1	Number of cases	VEGF (number of cases)		*r*	*p*-value
			Positive	Negative		
CNC	Positive	32	15.000	17.000	−0.327	0.006
	Negative	37	29.000	8.000		
BLBC	Positive	19	6.000	13.000	−0.332	0.021
	Negative	29	19.000	10.000		
Control group	Positive	26	13.000	13.000	−0.368	0.027
	Negative	10	9.000	1.000		

**Table 7:** Correlation analysis of HIF-1α and VEGF in CNC and BLBC and CNC and control group.

Groups	HIF-1α	Number of cases	VEGF (number of cases)		*r*	*p*-value
			Positive	Negative		
CNC	Positive	58	39.000	19.000	0.166	0.173
	Negative	11	5.000	6.000		
BLBC	Positive	28	18.000	10.000	0.374	0.009
	Negative	20	7.000	13.000		
Control group	Positive	19	16.000	3.000	0.501	0.002
	Negative	17	6.000	11.000		

In the BLBC group, the positive rate of VEGF was significantly higher in the BRCA1-negative group (65.51%) than in the BRCA1-positive group (31.57%), and the expression of VEGF and BRCA1 showed an inverse correlation (*r* = −0.332, *p* < 0.05). The positive rate of VEGF was significantly higher in the HIF-1α-positive group (67.85%) than in the HIF-1α-negative group (30.00%), and the two were positively correlated (*r* = 0.374, *p* < 0.05) (as depicted in **Tables 6**, **7**).

#### Analysis of the results of P53 and Ki-67 protein expressions

The positive rates of P53 expression in CNC and BLBC were 60.9% and 87.5%, respectively, and statistical differences were found between CNC and BLBC (*p* < 0.05); the high expression rates of Ki-67 in CNC and BLBC were 73.9% and 93.8%, respectively, and there were also statistical differences between the two (*p* < 0.05) (as depicted in **Table 8**).

**Table 8:** Comparison of P53 and Ki-67 expression differences between CNC and BLBC.

		CNC	BLBC	*χ* ^2^	*p*-value
P53	Positive	42 (60.9%)	42 (87.5%)	9.914	0.002
	Negative	27 (39.1%)	7 (12.5%)		
Ki-67	Positive	51 (73.9%)	45 (93.8%)	7.564	0.006
	Negative	18 (26.1%)	3 (6.3%)		

## Discussion

The existence of CNC as distinct breast cancer is quite characteristic in morphology. At present, there are only five large sample studies on CNC at home and abroad, and the research on the clinicopathological features of CNC is relatively scarce. In order to improve the understanding of clinicians and pathologists about the disease, the study investigated the relationship between 69 cases of CNC and 48 cases of BLBC. CNC was a concept first proposed by Jimenez et al. in 2001 (1), and they argued that CNC had the following four main characteristics: (1) The tumor was a single nodule type with a clear boundary. (2) The center of the tumor was accompanied by extensive necrosis (more than 70%), and the necrotic zone was usually accompanied by degenerative changes such as fibrosis and collagenization. (3) The necrotic zone was surrounded by residual cancer tissue distributed in ribbons. (4) Residual cancer tissues were often poorly differentiated and lacked glandular structure, presenting as high-grade invasive ductal carcinomas, the majority of which were accompanied by ductal carcinoma *in situ*. However, some scholars have suggested that the central necrotic area of more than 30% of the tumor area can be classified as CNC (11). To avoid the ambiguous concepts of CNC, this study selected cases with necrotic areas > 70%. BLBC first proposed that it was based on differential expression profiles of genes. Still, due to the high costs of genetic testing and complicated steps, it cannot easily be implemented in clinical work and has not yet become widespread. In 2004, Nielsen et al. proposed the use of immunohistochemical staining instead of genetic detection to classify molecular subtyping of breast cancer, defined a group of breast cancer cells expressing basal cytokeratins and/or EGFR as BLBC, and pointed out that more than 1% of tumor cells expressed CK5/6 and/or EGFR were the best options for diagnosing the BLBC (12).

### Comparison of clinical characteristics

CNC showed the preference of middle-aged and older women, with an average age of about 50 years old, an incidence rate of 2%–3%, rapid clinical progress, and strong invasive ability. Most of the previous reports were T1 and T2 tumors (85% in Tsuda’s report (11), 73% in Jimenez’s report (2), and 100% in Yu’s report (4)), and the status of axillary lymph nodes seems to be more negative (53% negative in Jimenez’s report (2), 64.5% negative in Zhang’s report (5)), while in Tsuda’s and Yu’s report, the positive ones are slightly higher (4, 11). In this group, the average age was 54.55 ± 9.683 years; both in T1 (40.6%) and T2 (59.4%) stages, 25 cases (36.23%) had axillary lymph node metastasis, 44 cases (63.77%) were negative, and the proportion of N1 stage was the highest (14.49%), similar to the above literature, and clinical stage II (39.1%) and pathological stage I (42.0%) accounted for the highest proportion. BLBC occurs mainly in premenopausal patients with an incidence of 10%–17%, and it is characterized by high malignancy, strong invasiveness, and a poor prognosis. In our study, the average age was 53.83 ± 11.372 years, and the tumor size staging results showed 17 cases (35.4%) in the T1 stage, 29 cases (60.4%) in the T2 stage, and two cases (4.2%) in the T3 stage; 21 cases (43.8%) had axillary lymph node metastasis, 27 cases (56.3%) were negative, and the proportion of N1 stage was the highest; and clinical stage II (43.8%) and pathological stage II (43.8%) accounted for the highest proportion. There were no significant differences in the clinicopathological parameters between the two groups (*p* > 0.05).

### Comparison of pathological characteristics

In general, CNC is a mostly single nodule with clear boundaries (ranging from 1.0 to 5.0 cm in diameter), and it is grayish-white in section with a slightly soft texture. The diameter of the CNC tumors in this group ranged from 1.2 to 5.0 cm, with an average of 2.55 ± 0.837 cm. Among them, two cases had two nodules in the ipsilateral breast and four cases showed unclear margins. Macroscopic observation of the HE slice of CNC showed that the obvious central pink staining area was wrapped by banded blue staining tissue. Under a light microscope, the central area was granular necrosis, which was surrounded by residual cancerous tissues distributed in a zonal pattern, and there were no transitional zones such as granulation tissue or fibrous tissue between the two components. The morphological features of necrosis were often of the following three types: (1) In the necrosis area, coagulative tumor necrosis was prevalent. (2) The central necrotic area was dominated by fibrillar collagen and glassy keloid-like tissue. (3) The necrosis was mainly infarction without fibrous collagen tissue, and a small amount of tissue outline can be seen in it. We point out that the fibrous, collagenous, cell-free zone in the center of the tumor is essentially a secondary change after tumor ischemia and necrosis. This is a dynamic evolution process: in the initial stage, it can be seen that there was only a small number of fibrous collagen bundles distributed in the coagulative necrosis-predominant background; as the tumor develops to the later stage, the secondary degenerative component of the necrotic area dominated, and a small amount of tumor cell residual ghosting was scattered in the glassy keloid-like background (4). The above three morphological features of necrosis were all observed in our study. We observed that the central cell-free zone showed coagulative tumor necrosis in 72.46% of cases, the cell-free zone presented fibrillar collagen and glassy keloid-like tissue in 27.54% of cases, and infarction in 13.04% of cases. However, it is not difficult for us to find that there were a small number of fibrous collagen bundles in the coagulative necrosis area and a small number of tumor cell residual ghosting in the context of extensive fibrous collagen. The surrounding residual cancer cells were poorly differentiated, with prominent nucleoli and vesicular nuclei; mitoses were common (>3/HPF) (3); and the glandular duct was lacking. In Jimenez’s report, the residual cancer cells can also appear as syncytial cell-like cells (1). Most residual cancer cells around the necrotic area were invasive ductal carcinoma in grade III. In the previous literature, cases with histological grade III accounted for 88.9%–91.8%, and cases with ductal carcinoma *in situ* accounted for 49.3%–63.6%. In the present study, 91.3% of the cases of peripheral cancer tissue were invasive ductal carcinoma in grade III, 8.7% were invasive ductal carcinoma in grade II, which was consistent with the literature, while the cases with ductal carcinoma *in situ* accounted for only 23.19%, which was inconsistent with the literature. Therefore, we speculate that the component of ductal carcinoma *in situ* may be explained by the residue of insufficiently thorough necrosis, and this group of cases were all CNCs with necrosis area > 70%, so it had a small proportion. In the previous literature, the residual cancer cells in a minority of cases can exhibit squamous and spindle cell differentiation, chondrometaplasia, invasive micropapillary carcinoma, mucinous carcinoma, intraductal papillary carcinoma, lymphocytic infiltrate, myxochondroid stroma, and calcification. In this group, three cases (4.35%) were focally differentiated into invasive micropapillary carcinoma, and myxoid stroma was observed in 14 cases (20.29%) (including one case of the chondrometaplasia). No squamous and spindle cell differentiation, mucinous carcinoma, or intraductal papillary carcinoma were observed. However, in the cancer stroma, 39 cases (56.52%) were accompanied by massive lymphocyte infiltration, 13 cases (18.84%) were accompanied by calcification, and one case (1.45%) was mainly infiltrated by plasma cells. The section of the BLBC was grayish-white, and the boundary was not clear. Under the microscope, an extensive cell-free zone was also visualized in the BLBC; necrosis was also common, usually in the form of focal, patchy, maze-like, or map-like lesions, and the edges were often pushing. Cancer cells showed poorer histological differentiation with significant pleomorphism, and most of them were histological grade III. The cancer tissues were basically free of glandular structure, often in nest-like, sheet-like, and diffuse distribution. In some cases, syncytial cell-like cells and squamous, spindle, and clear cell differentiation can be observed (13, 14). Carcinoma stroma was also often accompanied by a large number of lymphocyte infiltration. In this study, 29 cases (42.03%) in the BLBC group were accompanied by necrosis, including nine cases (31.03%) with a map-like or band-like shape of necrosis, three cases (10.34%) with scar-like fibrosis in the necrosis, and two cases (6.90%) with an infarction. A total of 46 cases (95.83%) were invasive ductal carcinoma in grade III, two cases (4.17%) were differentiated into grade II, and two cases (4.17%) were accompanied by intraductal carcinoma. In the cancer stroma, 18 cases (37.5%) were accompanied by profuse lymphocyte infiltration, three cases (6.25%) were accompanied by calcification, and one case (2.08%) contained areas of the myxoid component. Among the above histological characteristics, only the presence of ductal carcinoma *in situ* and mucinous stroma had statistical differences between CNC and BLBC. Based on the above literature review and observation of this group of cases, we found that CNC and BLBC have morphological similarities.

### Comparison of immunohistochemical characteristics

At present, information on the results of the CNC immunological phenotype is limited. In Tsuda’s report, expression of myoepithelial markers was observed in 80% of cases, and they point out that CNC may be caused by pathological differentiation of tissues to the myoepithelial direction or directly caused by myoepithelial carcinogenesis. However, Jimenez’s view leans more towards the former (1). In the reports of Yu and Zhang (4, 5), it was found that the expression rates of basal cell markers in CNC were 87.9% and 72.2%, respectively, and they pointed out that CNC was more likely derived from basal cells or pathological differentiation into basal cells. The expression rate of basal cell markers in this group was 66.67%, which was close to that reported in the literature but slightly lower than that reported in the previous literature. In our study, triple-negative breast cancer accounted for 53.6% and 93.8% of CNC and BLBC, respectively, and there was a significant statistical difference between them (*p* < 0.05), while in Yu’s series (4), the rate of triple-negative breast cancer was 66.7%, and this is slightly higher than our group. At present, only Yu’s and Zhang’s studies on molecular typing of breast cancer have been published, so there are few studies on CNC molecular typing. In the studies of Yu and Zhang (4, 5), the proportions of BLBC is 63.6% and 50.7%, respectively. In total, 56.52% of CNCs in our group are BLBC, which is consistent with the above results; that is, the BLBC subtype has the highest proportion.

BRCA1 is a tumor suppressor gene that can increase predisposition to tumorigenesis after mutation, and this is mainly reflected in breast and ovarian cancer. A total of 60%–90% of BRCA1 mutant breast cancers were BLBC, and genetic testing found that BRCA1 mutant breast cancer and BLBC display a high level of homology, and there were also obvious similarities in the expression of ER, PR, and HER-2 (15, 16). The National Comprehensive Cancer Network (NCCN) guidelines recommend detecting BRCA1/2 gene mutation for breast cancer patients of any type, which can guide the use of PARP inhibitors. In our study, the positive rates of BRCA1 in CNC and BLBC were 46.4% and 39.6%, respectively, and there were no statistical differences between the two (*p* > 0.05), while there was a significant statistical difference between CNC and the control group (*p* < 0.05). In this group, triple-negative breast cancer and BLBC accounted for 53.6% and 56.52% of CNC, respectively, and it can be seen that both of them account for a higher proportion in CNC, whether it was analyzed from triple-negative breast cancer or BLBC. Given the high proportion of triple-negative breast cancer in BLBC and the common feature of BRCA1-defective breast cancer and BLBC, perhaps for the treatment modalities of CNC, we can attempt to use targeted therapy for BRCA1.

It is currently not clear why CNC had such extensive necrosis. The presence of necrosis indicates that the tumor is insufficiently supplied with nutrients, and the supply of blood and oxygen as the basis for tumorigenesis and development is in close relationship to necrosis. Hypoxia can induce the expression of HIF-1α in response to hypoxia in the cellular microenvironment, and when hypoxia reaches a critical level, it will cause tumor cell necrosis, but usually, the area of necrosis does not exceed 30% (17). Large necrosis areas as a manifestation of CNC have attracted a wide attention of scholars. Currently, there is no information on the expression of HIF-1α in CNC. The present study demonstrated that the positive rates of HIF-1α expression in the CNC, BLBC, and control groups were 84.1%, 58.3%, and 52.8%, respectively, and there were significant statistical differences among the three groups (*p* < 0.05), but there was no statistical difference between the BLBC group and the control group (*p* > 0.05). From the above results, we point out that HIF-1α is expected to become a distinctive feature of CNC in some aspects. As has been reported in an earlier study, the HIF-1α pathway was hyperactivated in triple-negative breasts (18). Perhaps targeting HIF-1α would be quite effective in CNC therapy.

VEGF is a critical element of angiogenesis in cancer tissues and normal tissues. A large number of studies have found that hypoxia-induced HIF-1α can effectively promote transcriptional activation and expression of VEGF, providing a basis for tumor growth, local invasion, and distant metastasis (19, 20). Regarding the characteristic large central necrotic area in CNC, Jimenez argued that it may be caused by tumor growth occurring too rapidly without sufficient angiogenesis (1). In our study, the expression rates of VEGF in the CNC, BLBC, and control groups were 63.8%, 52.1%, and 61.6%, respectively, and there was no statistical difference among the three groups (*p* > 0.05). We also point out that large necrosis in CNC is related to insufficient angiogenesis.

Normally, BRCA1 can inhibit the activity of VEGF and reduce its secretion to impede tumor growth, invasion, and metastasis. However, when BRCA1 is mutated, VEGF will be overexpressed to promote tumorigenesis and tumor development (21). In this study, we analyzed the correlation between BRCA1, HIF-1α, and VEGF gene expression in CNC and BLBC. The results showed that BRCA1 and VEGF were negatively correlated in CNC, BLBC, and control groups (*p* < 0.05), and HIF-1α and VEGF were positively correlated in BLBC and the control group (*p* < 0.05), while in the CNC, the expression of VEGF in the HIF-1α-positive group was significantly higher than that in the HIF-1α-negative group, but there was no correlation between the two (*p* > 0.05). This result is consistent with Jimenez’s view that large necrotic areas in CNC may be caused by tumor growth occurring too rapidly without sufficient angiogenesis.

P53, as a tumor suppressor gene, is an expressed product that can inhibit tumorigenesis. When it mutates, its expressed product can be detected in tissues, which is called mutant P53. Our study demonstrated that the positive rates of mutant P53 in CNC and BLBC were 60.9% and 87.5%, respectively, and that there were significant statistical differences among them (*p* < 0.05). Ki-67 is a non-histone nuclear cortex protein, and it is located on chromosome 10q25-ter. Ki-67 is expressed in the cell nucleus during the G1, S, G2, and M phases of the cell cycle but not in the cell quiescent state, so it can serve as an alternative to the cell’s proliferative activity. In our study, the high expression rates of Ki-67 in CNC and BLBC were 73.9% and 93.8%, respectively, and statistical differences were found between CNC and BLBC (*p* < 0.05). The positive rates of P53 and Ki-67 in the BLBC group were higher than those in the CNC group, and the difference was statistically significant.

### Comparison of prognosis

The CNC has a high invasive capacity and rapid clinical progression. In previous literature, it has a high rate of recurrence and metastasis, and the most common metastatic sites are the lung and brain (1, 4, 5). In this group, 31 cases of CNC patients were followed up on, including nine cases of disease progression, five of which died, two cases of lung metastasis, one case of brain metastasis, and two cases of liver and bone metastasis at the same time. In our case, the brain metastasis rate was lower and was not the same as in the previous reports. In previous literature, the central necrosis area, tumor size, and lymph node status were considered independent prognostic factors for the disease (1, 4, 5). Zhang’s report also studied the relationship between necrosis area and basal cell markers and CNC recurrence and metastasis, but these results were not statistically significant (5). In our group, the analysis of the data demonstrated that age, tumor size, lymph node status, basal cell markers, histological grade, expression of VEGF, HIF-1α, and BRCA1, clinical stage, and pathological stage were not associated with disease progression (*p* > 0.05). However, this result might be related to fewer follow-up cases and shorter follow-up periods. BLBC usually has a poor prognosis, a higher metastasis rate, and is more prone to lung and brain metastases than other types of breast cancer. In our group, 20 cases of BLBC patients were followed up on, resulting in seven cases of disease progression, three of which died, one case of brain metastasis, and three cases of sternal metastasis. Lymph node status, clinical stage, and pathological stage were related to disease progression (*p* < 0.05), while other clinicopathological parameters and the expression of VEGF, BRCA1, and HIF-1α were not associated with disease progression (*p* > 0.05). The survival time of CNC was relatively shorter than that of BLBC, but it was not statistically significant (*p* > 0.05).

CNC has a high metastasis rate and a relatively poor prognosis (1, 2, 4). The most prominent feature of CNC is that the center of the tumor was accompanied by extensive necrosis, and the necrotic zone was usually surrounded by residual cancer tissue distributed in ribbons. Such a wide area of necrosis also suggests that CNC is more malignant and more aggressive (1, 22, 23). The Ki-67 index of residual cancer cells in the zonal distribution around the necrotic area was relatively low, which may be due to the existence of slow-cycling quiescent cells or senescent cells in these residual cancer cells. These tumor cells were identified as cells able to reactivate upon serial transplantation, survive chemotherapy and endure metabolic stress, and transform into stem-like cells with the ability of self-renewal, clonal evolution, and differentiation into new tumor cells (24). They can enhance the process of epithelial–mesenchymal transition, which provides a convenient pathway for cancer metastasis (25–27). It has been reported that hypoxia- and glucose metabolism-related pathways are activated and upregulated in slow-cycling quiescent cells (28). These cells induce the production of HIF-1α and form a new tumor microenvironment, the immunosuppressive tumor microenvironment, in which HIF-1α inhibits T-cell infiltration and increases T-cell exhaustion (28). The hypoxic tumor microenvironment also further increased the proportion of slow-cycling quiescent cells or senescent cells in breast cancer (26, 27, 29). In our study, the positive rate of HIF-1α in the CNC group was relatively higher than that in the BLBC group and the control group. The higher expression of HIF-1α in the CNC group may help slow-cycling quiescent cells or senescent cells to evade the attack of immune cells, and this mechanism may explain the poor prognosis of CNC patients. In future work, the more detailed mechanism still needs further study.

## Conclusions

At present, our understanding of the characteristics of CNC is inadequate. Although it has unique histological characteristics, its histopathological morphology and immunohistochemical phenotype are highly overlapping with BLBC, and the relationship with BLBC has not been completely unraveled.

In this study, the analysis of immunohistochemical results revealed that the expression of BRCA1 is similar in CNC and BLBC, and the targeted therapeutics to BRCA1 may also have beneficial potential in CNC; the expression of HIF-1α in CNC is significantly higher than that of BLBC, which may serve as novel entry points to distinguish between CNC and BLBC. There is a significant correlation between the expression of VEGF and HIF-1α in BLBC, and there is no correlation between the two in CNC. Therefore, we speculated that the large area of necrosis in CNC may be related to insufficient angiogenesis. However, the relationship between the two cannot be accurately described by immunohistochemical staining. In future work, gene detection should focus on comparing the similarities and differences of gene expression profiles between CNC and BLBC so as to further analyze the relationship between them.

## Data availability statement

The original contributions presented in the study are included in the article/supplementary material. Further inquiries can be directed to the corresponding author.

## Ethics statement

The studies involving human participants were reviewed and approved by Ethics Committee of the First Affiliated Hospital of Bengbu Medical College. The patients/participants provided their written informed consent to participate in this study. Written informed consent was obtained from the individual(s) for the publication of any potentially identifiable images or data included in this article.

## Author contributions

ZC studied concept and design. LD, WX, and SC acquired data and analyzed data. LD drafted the manuscript. WX and SC provided acquisition, analysis, and interpretation of data, and statistical analysis. All authors contributed to the article and approved the submitted version.
